# The community engagement course and action network: strengthening community and academic research partnerships to advance health equity

**DOI:** 10.3389/fpubh.2023.1114868

**Published:** 2023-06-19

**Authors:** Tabia Henry Akintobi, R. Barrett, L. Hoffman, S. Scott, K. Davis, T. Jones, N. De Veauuse Brown, M. Fraire, R. Fraire, J. Garner, A. Gruner, J. Hill, R. Meckel, C. Obi, P. Omunga, Q. Parham, T. Rice, O. Samples, T. Terrill

**Affiliations:** ^1^Morehouse School of Medicine Prevention Research Center, Atlanta, GA, United States; ^2^Morehouse School of Medicine Prevention Research Center Community Coalition Board, Atlanta, GA, United States; ^3^Georgia State University, School of Public Health, Atlanta, GA, United States; ^4^Coalition of Latino Leaders (CLILA), Dalton, GA, United States; ^5^Georgia Department of Natural Resources, Atlanta, GA, United States; ^6^Fort Valley State University College of Agriculture, Family Sciences and Technology, Fort Valley, GA, United States; ^7^Savannah State University, Political Science and Public Affairs, Savannah, GA, United States; ^8^Dalton State College Allied Health and Social Work, Dalton, GA, United States

**Keywords:** Community-based participatory research, community engaged research networks, collaboration, community-academic partnerships, marginalized populations, participatory evaluation

## Abstract

**Background:**

Historically Black Colleges and Universities and Minority Serving Institutions are uniquely positioned to implement community-campus research partnerships based on a history of service, the pursuit of community trustworthiness and student demographics often similar to surrounding marginalized communities. The Morehouse School of Medicine Prevention Research Center collaborates with members of Historically Black Colleges and Universities, Minority Serving Institutes, and community organizations on the Community Engaged Course and Action Network. This network is the first of its kind and aims to strengthen members’ ability to implement Community-Based Participatory Research (CBPR) principles and partnerships. Projects address public health priorities including mental health among communities of color, zoonotic disease prevention, and urban food deserts.

**Materials and methods:**

To assess the effectiveness of the network, a Participatory Evaluation framework was implemented to conduct process evaluation which included review of partnership structures, operations, project implementation processes, and preliminary outcomes of the research collaborations. A focus group of Community Engagement Course and Action Network members (community and academic) was also conducted to identify benefits and challenges of the network with emphasis on key areas for improvement to further enhance the relationships between partners and to facilitate their subsequent community-campus research.

**Results:**

Network improvements were tied to themes strengthening community-academic partnerships including sharing and fellowship, coalition building and collaboration, and greater connections and awareness of community needs through their current community-academic partnerships. The need to conduct ongoing evaluation during and after implementation, for determining the early adoption of CBPR approaches was also identified.

**Conclusion:**

Evaluation of the network’s processes, infrastructure, and operation provides early lessons learned to strengthen the network. Ongoing assessment is also essential for ensuring continuous quality improvement across partnerships such as determining CBPR fidelity, assessing partnership synergy, and dynamics, and for quality improvement of research protocol. The implications and potential for advancing implementation science through this and similar networks are great towards advancing leadership in modeling how foundations in community service can advance to CBPR partnership formation and ultimately, health equity approaches, that are local defined and assessed.

## Introduction

Community engaged research is defined as the process of working collaboratively with and through groups of people affiliated by geographic proximity, special interest, or similar situations to address issues affecting the wellbeing of those people (1, p. 7). It is focused on building and sustaining strong community-academic partnerships and operates along a continuum of increasing community involvement in research activities. Community-based participatory research (CBPR) is a type of community engaged research, emphasizing equitable community engagement in research. Historically Black Colleges or Universities and Minority Serving Institutions are uniquely positioned to implement effective CBPR based on a history of service and the pursuit of community trustworthiness central to their missions.

The Community Engaged Course and Action Network was designed to establish a capacity building, CBPR infrastructure for and by Historically Black Colleges and Universities and Minority Serving Institutions adapting an established CBPR model led by Morehouse School of Medicine. Most of these schools are entirely integrated into their surrounding communities (e.g., most students are commuters especially at the public institutions) often with shared social, cultural values, behaviors and demographics of surrounding marginalized communities. It is no coincidence that rural areas and the portions of the Micropolitan Statistical Areas containing historically black colleges or universities are also some of the most underserved areas in terms of healthcare accessibility and inversely these areas experience high rates of poverty and income inequality. Thus, there is a great need and potential to educate faculty at these institutions to learn and model CBPR partnerships to address local health priorities for the future public health leaders at these institutions.

Partnership and administrative factors are assessed through a Participatory Evaluation framework. This approach has been previously employed by the Morehouse School of Medicine Prevention Research Center to equitably and strategically, engage project implementation partners and stakeholders due to the welcome complexities of CBPR and historical power inequities detailed earlier. We seek to facilitate a supportive network towards advancing related partnerships in response to local health priorities for a network of newly partnered collaborators. Participatory Evaluation approaches are designed to address needs mutually identified by partners to assure that initiatives are: (1) audience-driven; (2) foster sustained ownership of evaluation processes; and (3) are central to program decision-making and sustainability (2). This also provides accountabilities to sponsors towards ensuring that recommendations, concerns, and preferences are heard towards sustaining program strengths and making quality improvements of identified challenges or weaknesses. Hence, the purpose of this study is to evaluate the community-academic partnership and research administration using process evaluation techniques.

## Background

Morehouse School of Medicine is one of ten Historically Black Colleges and Universities in Georgia and one of only four Historically Black Medical Colleges in the United States. Morehouse School of Medicine holds over four decades in globally recognized implementation of a cross-cutting (clinical, research education and service) community-centered vision - *to lead the creation and advancement of health equity* (3). The Morehouse School of Medicine Prevention Research Center is the designated center for CBPR within the institution. For over two decades, through collaborative relationships with community members and organizations, Morehouse School of Medicine Prevention Research Center has been competitively funded to conduct applied to HIV/AIDS, substance abuse, cardiovascular, diabetes, oral health, behavioral, clinical and translational research as well as lead related research infrastructure and capacity building efforts (4–17). The Morehouse School of Medicine Prevention Resource Center has collaborated with its Community Coalition Board to build trusting and mutually beneficial relationships with communities. The Center is governed by the Board, which was established in 1999 to articulate community priorities and advance health equity (10, 18–21).

The Community Engagement Course and Action Network, established in 2019, is an extension of the Morehouse School of Medicine Prevention Research Center mission to strengthen community engagement, research, leadership and partnership models designed to innovatively, advance health equity through strategically identified potential partner communities. The network was established to strengthen the capacity of minority serving institutions to develop locally responsive community-campus CBPR partnerships. The goal of the network is to strengthen the ability of Historically Black Colleges and Universities, Minority Serving Institutions, public health agencies, medical practitioners, students, and multi-sector partners in CBPR and community engaged research.

## Materials and methods

### Community engagement course and action network

#### Structure

The network began with 10 Georgia Historically Black Colleges and Universities and Minority Serving Institutions when the network was launched in May 2020. Due to the global COVID-19 pandemic and other factors, four Georgia academic institutions remained committed to working with MSM. These included: Dalton State College, Fort Valley State University, Georgia State University, and Savannah State University. Table 1 details the health conditions in communities in the counties surrounding each school supporting the rationale to offer intensive outreach, education and capacity building to Historically Black Colleges and Universities, Minority Serving Institutions, and partnering communities towards applied public health prevention research and implementation strategies. The Center launched a mini-grant program to support community-university research partnerships, facilitate community input into university research, and to increase health research in community settings that is both responsive and relevant to the health needs of the community. Projects, partners and focus areas are detailed in Tables 2,3.

**Table 1:** Socioeconomic characteristics of CECAN research partner counties.

CECAN partner counties and sites	Population living below federal poverty level (%)*	Minority population (%)*	Proportion of population with less than HS diploma*	Number of primary care physicians per population**	Population with low access to store (%)***
Chatham County (Savannah State University)	17.9	46.7	8.8	1:1,130	22.1
Fulton County (Georgia State University)	16.9	54.8	6.0	1:573	23.5
Peach County (Fort Valley State University)	21.0	51.9	13.2	1:1,820	19.4
Whitfield County (Dalton State University)	15.0	39.0	20.3	1:3,424	NA
Georgia	17.8	40.2	10.7	1:1,30	NA

* Data source: National Historical Geographic Information System. American Community Survey Summary Files (2020). Retrieved from: https://www.nhgis.org/.

** Data source: Primary Care Practitioners Workforce Projections. Health Resources and Services Administration (December 2020).

*** Data Source: United States Department of Agriculture. Food Atlas (2019). Retrieved from: https://www.ers.usda.gov/topics/food-choices-health/food-access/.

Low access to store: people in a county living more than 1 mile from a supermarket or large grocery store if in an urban area, or more than 10 miles from a supermarket or large grocery store if in a rural area.

**Table 2:** CECAN research partners and projects.

Developing Culturally Appropriate Mental Health Network for Latinx Community – Dalton State College (DSC), the first college in the University System of Georgia designated as Hispanic Serving Institution, partners with the Coalicion de Lideres Latinos (The Coalition of Latin Leaders), an all-volunteer local organization to reach the Hispanic residents in Dalton County, to address mental health concerns. The partnership is still being developed as the academic role has been redefined and the community-based organization role has been amplified
Mental Health Outreach through Black Barbers (The Confess Project - TCP) – Georgia State University, the largest in the University System of Georgia and designated as a Minority Serving Institution, is examining the impact that participating in TCP has on barbers’ knowledge, attitudes/beliefs, skills, and behaviors related to mental health. TCP is “America’s First Mental Health Barbershop Movement,” which began in 2016 to address the need for programming that can equip African American men with knowledge, strategies, and coping skills to help them recognize and address mental health challenges. The data collected provides a better understanding of barbers’ views, perceptions, and attitudes about mental health since these can influence their capacity and willingness to be mental health advocates for their clients and communities. Further, baseline and follow-up data on the barbers is being collected for the first time. Therefore, the evaluation will generate useful information that will help identify areas of the training/model that should be modified to improve the curriculum based on the data collected and to achieve the project’s anticipated outcomes. Specifically, the pilot study aims to answer an important question about TCP: can TCP-trained barbers help reduce mental health stigma among black men/boys and positively impact their mental health?
Prevention of Zoonotic Disease Transmission – Fort Valley State University (FVSU), the only university in the world that is concurrently a University System of Georgia Institution, a Historically Black College or University (HCBU) and a land grant institution, partners with the Department of Natural Resources (DNR) within Burke County, Georgia to provide training to technicians of licensed Doctor of Veterinary Medicine (DVM) and Veterinary Technicians on proper field necropsy techniques to determine zoonotic disease potential in wildlife species. Zoonotic diseases that interface between wildlife and outdoors enthusiasts (hunters, taxidermists, game processors) remain an under-addressed topic in mainstream public health and yet the threat is real. Natural resource technicians are often called upon when wildlife carcasses present either in general population environments (yards, farms, etc.) or on state/federal lands (WMA’s, parks, etc.) to provide answers and assistance in both removal and protection of human populations sharing these areas. With the prevalence of such zoonotic diseases as rabies, salmonella, or presence of certain parasite vectors capable of spreading disease between carcass and humans, the DNR technician is the first line of defense in monitoring the status of indigenous wildlife populations. Training of Department of Natural Resources technicians their knowledge of zoonotic disease transmission and safe necropsy procedures and provide printed necropsy guide for technicians that may be shared throughout the agency to ensure all technicians education on safe handling of wildlife carcasses during field work
Addressing the Challenges of Urban Food Deserts – Savanah State University (SSU), the oldest public historically black college or university, partners with a Community-based organization the Harambee House in Savanah, GA to implement community engagement at Harambee House will be focused on community education and outreach that leverage on building capacity and community support networks, that will help potential urban farmers and urban community residents to assess and better understand the existing urban land use practices and innovative entrepreneurial agriculture opportunities, discover connections, and establish shared vision among practitioners. The participating community was educated on the unhealthy impact of their limited local food resources, and the various ways they can augment their dietary choices and improve the availability of food and groceries in their neighborhood.

**Table 3:** CECAN prioritized populations/communities.

Prioritized populations/communities	History of population inequality	Sample health disparities	Root cause of vulnerability
Latina/o and Hispanic	Immigrant status Mass incarceration Language barriers	Brown versus white life expectancy Mental health stigma	Historical mistrust Wage/wealth gaps
African American men	Slavery Mass incarceration police brutality	Black versus white life expectancy Access to appropriate mental health service	Structural racism Discrimination Medical mistrust Wage/wealth gaps Mental health stigma
Department of Natural Resources Technicians and Wildlife Hunters	Under-resourced workers	Covid-19 healthcare workers and use of PPEs	Essential workers’ health and safety
Underserved Rural Communities	Barriers and health and healthcare services	Higher prevalence of risk factors for Chronic diseases	Poverty Food insecurity Inadequate transportation Lack of medical providers

#### Operations

To support community leadership, the network is led by the chair of the Morehouse School of Medicine Prevention Research Center Community Coalition Board. Essential to the Center’s mission, the Board chair has been community and research identified as a community developer, civic engagement practitioner, and health advocate. His work ensures community-centered, led and translated health equity leadership and governance to support network members. He is a community economic development practitioner and long-time Morehouse School of Medicine community partner. His leadership with and for diverse groups of community advocates, academic partners, and agencies in strategies to achieve equity and enhance the built and natural environment that reduce health disparities and promote community-campus partnerships using the CBPR makes him a valued leader of the network. He is supported by staff of the Center’s to facilitate meetings, convening, communication, and evaluation processes.

A qualitative assessment of the community-academic partnership was conducted by reviewing the Community Engagement Course and Action Network establishment, structure, partnership dynamics, and outcomes of partnerships based on the alignment with the CBPR principles. A review of partnership formulation documents, funding structures, and reports from meetings, webinars, technical assistance and support, and workshops provided additional contextual data for assessment of the network partnership dynamics. In addition, a focus group (*N* = 12) of Community Engagement Course and Action Network members (both community and academic partners) was conducted face-to-face and supported by Mentimeter, a survey polling tool (22). The focus group was designed to identify benefits and challenges of the network with emphasis on key areas for improvements to further enhance the relationships between partners and to grow the network. The group, together, identified areas of success and areas for improvements in creating synergy and cohesiveness within the network. This PE framework was essential to ensuring that *all* community-campus partners involved in the network share and hear the similar and disparate issues central to their participation and perceived network success. This was critical given the CBPR network being facilitated and the well understood power differentials address through CBPR that the network is designed to dismantle and collaboratively support towards responsive, partners community response.

Process evaluation involved review of partnership documents aforementioned. Focus group data analysis was manual and involved two coders that developed an initial codebook informed by questions guiding the focus groups discussion. Summary points were transferred into.

a matrix to systematically code responses. Emerging codes were noted and included in the final coding guide (23). Themes associated with codes were reviewed by the Center’s Community Coalition Board Chair (network facilitator) and Center staff and identified detailed in the section that follows.

## Results

### Key themes

The findings of the qualitative assessment of Community Engagement Course and Action Network research partnership were subdivided into sections, in relation to commonly identified themes, to summarize the outcomes of the collaboration between partners based on the CBPR framework.

### Building and strengthening partnership

Despite the well-documented benefits of CBPR [(24, 25); Jagosh, et al., 2012], there are challenges in building and maintaining community-academic research partnerships to improve community health outcomes. It is critical before establishing community-academic partnerships for CBPR to first find out the issues that are affecting the community and understand the interest of the community. Georgia State University CECAN research project is a testament of academic partners working collaboratively with the community to overcome challenges in conducting research in barbershops and barber schools across metro Atlanta. After listening to these community partners and valuing their contributions, the university designed a pilot study aimed at appropriately evaluating and improving a novel program that trains barbers and barber students to be mental health advocates. Considerable formative work went into establishing and strengthening a community-academic partnership well before the research began, and these efforts continue throughout project (i.e., “cradle to grave”) to ensure that the community’s voice, ideas, and concerns are foundational to this collective work. Through focus group discussions with this community of interest (i.e., barbers, barber students, and shop owners) – as well as participant surveys and barber advisory board input – the partners were able to collect process and outcome data that will be used to enhance the training program. Ultimately, the program will better meet the needs and interests of barbers trained to serve as mental health advocates in their communities. Researchers should approach partnership within communities with honest intentions and humility, recognizing that building such relationships takes time and unwavering efforts over the long-term.

### Establishing authentic partner engagement

Effective evaluation of partnership efforts requires a clear conceptual framework that links group dynamic characteristics of equitable partnerships (e.g., shared leadership, meaningful participation, and power sharing) with the effectiveness of partnership efforts to intervene and reduce health inequities more broadly (26). For example, for the networks research project, *Developing Culturally Appropriate Mental Health Network for Latino/a Community*, Dalton State College worked with Coalicion de Lideres Latinos a Latino/a grassroot community advocate group, to assess and identify the need for mental health services within the Latino/a community. Coalicion de Lideres Latino/a assumed the role of cultural broker to continue building trust and rapport with community members. Researchers should collaborate with community partners to ensure that that they are recognized and valued within the community as a trusted agent of change. Being present in the community helps to build trust and commitment to engagement and ultimately establish co-leadership for research.

### Network synergy and expansion

A critical dimension in many CBPR frameworks is the concept of partnership synergy, or synergy that arises from collaboration among members of diverse knowledge, perspectives, cultures, and social positions (27). Synergy is the concept of gainfully accomplishing more collaboratively, opposed to separately which is critical to partnership effectiveness and expansion. For example, Savannah State University collaborated with their community partner, Harambee House to address the challenges of urban food desserts in underserved rural communities, while embracing the idea of a community-led project approach. Harambee House is well-known for promoting civic engagement, environmental justice, and social change but needed further guidance on how to properly execute CBPR. The partnership was initially hampered because the responsibilities of both partners were not clearly defined or based on the expertise of each partner. For the second round of funding, the community partner, instead of the academic institution, became the lead, which resulted in a delay in implementation of the research project causing both partners to reevaluate their roles and responsibilities. In CBPR, addressing the structural, social, and cultural differences between community and academic is critical for successful implementation of research projects. Sharing knowledge, expertise, decision-making, capacity building of community partners, and other resources would help in breaking down silos and strengthening unity for the success of such partnerships.

### Institutional and leadership support

Researchers invested in community– academic partnership requires institutional and leadership support to be successful (28). This level of partnership investment requires commitment in creating an environment of co-leading, co-learning, and capacity building that is beneficial for all members of the partnership (29). While the Community Engagement Course and Action Network research projects were well supported by academic researchers, there is limited knowledge of whether the community-academic partnership was supported by each university/college academic leadership. Morehouse School of Medicine Prevention Research Center’s leadership fully embraced the use of CBPR model and expressed its commitment to the establishment of community-academic partnerships for the Community Engagement Course and Action Network research projects by contracting with its Community Coalition Board chair, a non-academic leader, to serve as a coordinator and facilitator for community engagement. The Community Coalition Board chair has 15 years of experience working with diverse groups of community advocates, academic partners, and agencies to develop strategies aimed at reducing health disparities and promoting community-academic research partnerships using the CBPR framework and a reflection of the leadership model with the Morehouse School of Medicine Prevention Research Center. This leadership structure allows for power-sharing between community and academia by building on existing trusting relationships as promoted by the Morehouse Model for Community Engagement. The Morehouse School of Medicine Prevention Research Center leadership team also offers technical assistance to the network research partners in areas of project administration, community partnership engagement, evaluation, and research translation and dissemination.

### Partnership and network engagement

One benefit of community engagement in research is gathering valid and credible evidence of diseases and designing meaningful and impactful culturally appropriate interventions based on the lived experiences of affected community members. The Fort Valley State University researchers admitted that their research on zoonotic diseases was strengthened by directly involving Department of Natural Resources technicians who were at increased risk for exposure to pathogens of the diseases and who had practical solutions for disease prevention and control. Upon conducting a 2022 focus group discussion among Community Engagement Course and Action Network research partners, we learned the expectations and benefits from both the academic and community-based organizations of participating in the network. Expectations and benefits coincided to include “*sharing and fellowship, coalition building and collaboration, and greater connections and awareness of community needs*,” (see Table 4). Community Engagement Course and Action Network research partners also expressed some of the successes (wins) they achieved through the network and recommendations for improving their project plans to strengthen the community-engaged approach. One of the most salient wins was “*bringing national attention to the concept of community engaged research at undergraduate level*,” (see Table 5). Recommendations for Community Engagement Course and Action Network improvements were tied to lessons learned from their current community-academic partnerships.

**Table 4:** CECAN partner reasons for joining and benefits of the network.

Questions	Responses
Why did you choose to join the CECAN Network?	Application of community – based research and applied research Sharing and fellowship Partnership engagement - community engagement Coalition building and collaboration Opportunities to learn, work collectively, and have impact Accessing extramural funding for research Establishing credibility in research and gain exposure for work Assurance of information credibility and data collection in research from a trusted academic institution
What do you believe are among the biggest benefits of participation in the network?	Opportunities to see value in the community – campus partnerships and to partner with community Opportunities to partner with community - actualized public health intervention by working in partnership with those who are adversely affected Access to expertise and information of Morehouse School of Medicine New opportunities and crowdsourcing of ideas Collaborations with organizations from different regions of Georgia Greater connections and awareness of community needs, greater influence on community through enhanced ability to conduct community-based research in an efficient way Sustainable relationship between academia and community in research Addressing community needs through collaborative engaged research Opportunity to have evidence to make informed decisions Being able to discuss challenges and having other organizations being able to provide relatable suggestions Gaining new perspectives in research

**Table 5:** CECAN partnership successes and opportunity for project improvement.

Questions	Responses
If you have experienced any early wins, what have they been?	Existing and potential partners Cementing or firming up my partnerships through gathering regularly Having access to this network Opportunity to project expansion Bringing national attention to the concept of community engaged research at undergraduate level Continued engagement in the network Opportunities for increased involvement of institution in community
In what ways have you had to adjust your project plans and why?	Reevaluate partners contributions to accommodate valuable community engagement Focus is a new endeavor rather than continuation of round one-but that’s a positive pivot Adjust how projects are being implemented and in turn how to move forward and tailor research/evaluation

## Discussion

Evaluation of the Community Engagement Course and Action Network research partnership facilitated an assessment of fidelity in application of the CBPR principles. By conducting a qualitative review of partnership structure, project implementation processes, and outcomes of the research collaboration, it was clear that consistent monitoring and technical assistance is essential for success of community-academic partnerships. There was evidence to support the claim that breakdown in communication between partners can result in undefined expectations and delay in the execution of research processes. Focus group discussions among the network’s partners did reveal some positive results regarding the benefits of the network with key areas for improvements to further enhance the relationships between partners and to grow the network. Noteworthy in informing other initiatives is that CBPR capacity building and partnership development support towards community-driven intervention, is the goal of the network. The focus of this manuscript was process evaluation of the network function and CBPR partnership formation to date. As the network and funded projects evolve (projects and processes are still underway) CBPR principles, in practice, will be comprehensively assessed.

The qualitative data provided great insights into the challenges that were experienced by the Community Engagement Course and Action Network research partners from which to learn and grow the network for the next phase of project implementation. One of the most important lessons learned is the need to conduct ongoing evaluation of networks collaboration efforts to improve synergy among community-academic partners and for effective project functioning. Establishing clear roles and responsibilities to ensure accountability for governance of the research project processes, including invoicing and reporting. Engaging community partners during the selection of pertinent issues prior to project developing and planning phase is critical to ensure that community needs are being prioritized in research. Building relationships with community partners prior to the research also allows for equal distribution of power between the community and academia, consistent with the co-leadership principle of CBPR. From the focus group discussion, academic partners are aware of the importance of engaging community partners and that their level of contributions will determine the success of the research project. However, the timeline for engagement should be clearly defined and the process of engagement needs to be better streamlined (Table 6).

**Table 6:** Recommendations for CECAN project and network enhancements.

Questions	Responses
What are the changes you recommend as we continue our work together?	Longer lead times-turning around contracts/invoices/etc. Everything works very well and cannot think of changes that need to be made Visiting the various sites to see the work in-person – was not possible during Covid-19 Getting to know our partners beyond the project; being creative and adjustable to changes; respect and trust building Opportunities to hear what is going on with other projects on a regular basis Mentor some community organizations on a deeper level to be more efficient
What are some recommendations for things you believe we can do as a network?	Seek funding as a group to support our collaborative work Developing student ambassadors to continue efforts Use the power of the network to help various projects find more funding to grow and expand on the CECAN projects Sharing knowledge and skills gained, building capacity - next generation of community-academic partnerships Have a dedicated website (do we already have this?) that showcases each project and drives interested readers to the project and health promotion of interest Representation at the Georgia Public Health Association to present projects Participate in Strategic Management classes for local organizations in their communities Engage with policy makers for project sustainability

To our knowledge, this is the first is the first network of Historically Black Colleges and Universities and Minority Serving centered on increasing CBPR capacities and conjointly providing funding to support skills and partnership acquired. Second a nationally recognized community leader, rather than Morehouse School of Medicine Prevention Research Center facilitating this network and partners were led by the chair of the Community Coalition Board at Morehouse School of Medicine Prevention Research Center collaborated with underserved communities and academic leaders representing unique priority population groups within the urban and rural areas of Georgia. We conducted a formative, participatory process evaluation study amongst network partners, both community and campus-based, who served as equitable experts on relevant community and partnership priorities and challenges.

A more comprehensive assessment of the community-academic partnership is warranted. Baseline data at the initiation of the Community Engagement Course and Action Network regarding the partnership structure, application of the CBPR principles in partnership, research design, and implementation, coupled with ongoing partnership evaluation would reveal areas of growth and areas for improvement over time. Conducting a partnership assessment after the mid-term implementation of the network’s research projects provided only a snapshot of the successes and challenges that were achieved over the two funding cycles. Nonetheless, the qualitative data collected on partnership dynamics can be used to enhance collaboration and strengthen cohesiveness between members of the network as it continues to progress.

The Morehouse CBPR Model with the complementary tools promotes collective reflection that, among other constructs, leads to our central theory of change—collective empowerment (30). Reflecting on the partnerships and the projects that Community Engagement Course and Action Network partners implemented, the research partners were asked about changes they would make to their projects and the network based on lessons learned and to develop next steps for enhancement of the partnership and network, (see Table 5). Recommendations were aimed at building/strengthening community-academic partnerships and increasing the legitimacy of the network’s research projects among researchers, communities (local, regional, and statewide), funders, policymakers, and other stakeholders.

## Conclusion

It is essential to assess community-academic partnership at initiation, during, and after research project implementation to determine its alignment with CBPR principles and to ensure continuous quality improvement across partnerships in a myriad of contexts. The results of such ongoing evaluation can be used to develop strategies to enhance partnership functions and to strengthen the cohesiveness of the partners within the network. The unique history and position of Historically Black Colleges and Universities and Minority Serving Institutions present opportunities to understand the conditions through with CBPR partnership development despite cross-cutting dedication to community trust, take place. The heterogeneity of institutions require attention to the contexts in which community-campus partnerships are successful, given differences in identity (private/public, teaching/academic health center, geography, and track record of relationship and power sharing among community-campus partners). The implications and potential for advancing implementation science through this and similar networks are great towards advancing leadership in modeling how foundations in community service can advance to CBPR partnership formation and ultimately, health equity approaches, that are local defined and assessed. Collaboration amongst Community Engagement Course and Action Network partners will expand the influence of Historically Black Colleges and Universities and Minority Serving Institutions which is vital for sharing ideas, knowledge, expertise, and other resources that advance their collective impact as strategic public health change agents.

## Data availability statement

The original contributions presented in the study are included in the article/supplementary material, further inquiries can be directed to the corresponding author.

## Author contributions

THA, RB, and LH: conceptualization. THA, RB, LH, and SS: writing—original draft development. THA, RB, LH, and TJ: designed research questions, and analysis tools. THA, RB, LH, and SS: conducted data analysis. THA, RB, LH, SS, NDB, MF, RF, AG, PO, QP, TR, OS, and TT: prepared original manuscript, and revised draft of manuscript. THA, RB, LH, SS, KD, TJ, NDB, NF, RF, AG, JH, RM, CO, PO, QP, OS, and TT: contributed to data collection, reviewing, and editing of revised manuscript drafts. All authors contributed to the article and approved the submitted version.

## Funding

This project was supported through funding from the Centers for Disease Control and Prevention (Grant # U48DP006411) and the National Center for Advancing Translational Sciences of the National Institutes of Health (Grant #UL1TR002378).

## Conflict of interest

The authors declare that the research was conducted in the absence of any commercial or financial relationships that could be construed as a potential conflict of interest.

## Publisher’s note

All claims expressed in this article are solely those of the authors and do not necessarily represent those of their affiliated organizations, or those of the publisher, the editors and the reviewers. Any product that may be evaluated in this article, or claim that may be made by its manufacturer, is not guaranteed or endorsed by the publisher.
